# *In Vivo* Imaging of Hedgehog Pathway Activation with a Nuclear Fluorescent Reporter

**DOI:** 10.1371/journal.pone.0103661

**Published:** 2014-07-28

**Authors:** John K. Mich, Alexander Y. Payumo, Paul G. Rack, James K. Chen

**Affiliations:** 1 Department of Biochemistry, Stanford University School of Medicine, Stanford, California, United States of America. Current address: Children's Research Institute, University of Texas-Southwestern Medical Center, Dallas, Texas, United States of America; 2 Department of Chemical and Systems Biology, Stanford University School of Medicine, Stanford, California, United States of America; 3 Department of Developmental Biology, Stanford University School of Medicine, Stanford, California, United States of America; Institute of Molecular and Cell Biology, Singapore

## Abstract

The Hedgehog (Hh) pathway is essential for embryonic development and tissue regeneration, and its dysregulation can lead to birth defects and tumorigenesis. Understanding how this signaling mechanism contributes to these processes would benefit from an ability to visualize Hedgehog pathway activity in live organisms, in real time, and with single-cell resolution. We report here the generation of transgenic zebrafish lines that express nuclear-localized mCherry fluorescent protein in a Gli transcription factor-dependent manner. As demonstrated by chemical and genetic perturbations, these lines faithfully report Hedgehog pathway state in individual cells and with high detection sensitivity. They will be valuable tools for studying dynamic Gli-dependent processes in vertebrates and for identifying new chemical and genetic regulators of the Hh pathway.

## Introduction

Zebrafish have emerged as versatile models of vertebrate biology, due to their amenability to genetic and pharmacological manipulations, optical transparency during embryogenesis and larval development, and facile and economical husbandry [1]–[4]. They have been used extensively to investigate the molecular and cellular mechanisms that contribute to tissue patterning [5] and more recently have contributed to our understanding of tissue regeneration, tumorigenesis, metabolism, infectious disease, and behavior [6]–[11]. As the importance of teleost models in biomedical research continues to grow, transgenic lines that can provide real-time indicators of specific biological events will be increasingly valuable. Accordingly, zebrafish can be readily engineered to express fluorescent reporters in selected tissues or upon the activation of individual cellular pathways [4].

*In vivo* reporters of developmental pathways are particularly instrumental, given the pervasive role of these signaling mechanisms in vertebrate biology. Several zebrafish lines that allow the real-time observation of cellular responses to Wnt, Hedgehog (Hh), bone morphogenetic protein (BMP), and fibroblast growth factor (FGF) family members have been described [12]–[19]. Each of these zebrafish lines utilizes a fluorescent protein reporter driven by *cis*-regulatory elements specific to the signaling pathway of interest. While these transgenic animals can reveal tissue-specific differences in cell signaling, the biological properties of their reporters can limit their utility. All but one of these lines utilizes fluorescent reporters without subcellular targeting motifs, and the cytoplasmic distribution of these proteins decreases their detection sensitivity. As a result, the reporters typically cannot be observed by fluorescence microscopy until several hours after their transcription commences, especially if destabilized versions are utilized to improve reporter dynamics. Second, although cell-wide dispersion of the fluorescence signal can provide useful, collateral information on cell morphology, it can also obscure differences in pathway activity among neighboring cells.

The ability of zebrafish with Gli-dependent enhanced green fluorescent protein (EGFP) [15], mCherry [16], or Kaede reporters [17] to emulate endogenous Hh pathway activity illustrates the utility and limitations of these transgenic lines. Zebrafish have four Hh isoforms, which have been classified as Sonic Hh (Shha and Shhb) and Indian Hh (Ihha and Ihhb) family members [20]–[22]. During the first 24 hours post fertilization (hpf), *shha*, *shhb*, and *ihhb* are expressed in distinct but overlapping domains, with transcripts initially detected within the dorsal mesoderm shortly after the onset of gastrulation (60% epiboly, 7 hpf). Hh ligand expression is restricted to the axial mesoderm as convergent extension proceeds, and these morphogens are produced by the developing notochord, medial floor plate, and ventral floor of the brain during somitogenesis. Zebrafish cells respond to Hh proteins through the Patched family of 12-transmembrane receptors (Ptch1 and Ptch2) [23], [24], leading to the activation of Smoothened (Smo) [25], [26], a G protein-coupled receptor-like component, and Gli transcription factors (Gli1, Gli2a, Gli2b, and Gli3) [27]–[30]. The degree and duration of Hh pathway activation in these cells then regulates their differentiation. For example, somitic tissues form slow-twitch muscle fibers and muscle pioneer cells in response to moderate and high levels of notochord-derived Hh signals, respectively [31]. In contrast to these endogenous processes, the previously described EGFP and mCherry reporter lines do not exhibit Gli-dependent fluorescence until mid-somitogenesis (approximately 17 hpf) [15], [16]. Nor can they clearly resolve the differences in Hh pathway activity that give rise to distinct muscle cell types during somite development. The photoconvertible Kaede line can be used to distinguish cell populations with temporally distinct Hh responses; however, the cytoplasmic reporter makes it challenging to differentiate cells with similar Hh signaling dynamics [17].

Targeting fluorescent reporter signals to the nucleus can help overcome these limitations, providing transgenic lines with improved signal-to-noise properties and single-cell resolution. *In vivo* reporters of Wnt signaling have been enhanced in this manner [14], and we therefore sought to establish new zebrafish lines that carry fluorescent, nuclear-localized reporters of Hh pathway state. Such genetically modified organisms could reveal how Hh signaling dynamically regulates formation of the brain, neural tube, somites, fins, and other tissues during zebrafish embryogenesis. Hh pathway activation also contributes to the regeneration of body parts such as a caudal fin [32], [33], and its dysregulation can promote the onset and/or maintenance of certain cancers [34].

We have generated two Hh pathway reporter lines: one that expresses mCherry functionalized with a nuclear localization sequence (NLS) upon Gli activation and another that carries a destabilized form of the mCherry-NLS reporter. Gli-dependent mCherry fluorescence is clearly evident in these transgenic animals by the one-somite stage (11 hpf), and divergent responses to Hh signaling can be observed in these transgenic lines with single-cell resolution. Genetic and chemical perturbations further confirm that the fluorescent signals in these lines faithfully communicate Hh pathway state, and the reporter is functional in both embryos and adult fish. We anticipate that these transgenic lines will be valuable tools for studying Hh pathway-dependent development and physiology. They could also facilitate the discovery of new signaling components and small-molecule modulators, taking advantage of the zebrafish's amenability to genetic and chemical screens.

## Materials and Methods

### Ethics Statement

All experiments involving zebrafish were approved by the Institutional Animal Care and Use Committee at Stanford University (Protocol ID: 10511).

### Zebrafish husbandry

Wildtype AB, *shha^t4^* [35], and *smo^hi1640^* [26] zebrafish were obtained from the Zebrafish International Resource Center, *dzip1^tm79a^* mutants [36] from Dr. Will Talbot, *gli1^ts269^* [36] mutants from Dr. Rolf Karlstrom, and *Tg(-2.4shha:GFP-ABC)* zebrafish [37] from Dr. Uwe Strähle. All zebrafish lines were maintained under standard conditions at 28°C.

### Generation of transgenic zebrafish

Transgenic fish were created through Tol2-based transgenesis as previously described [38]. Each transgenesis vector was generated through the following cloning steps:

#### 8xGliBS-EGFP-Odc1

8xGliBS-Luciferase [39] was digested with KpnI/NcoI to release the 8xGliBS-δ-crystallin minimal promoter cassette. This fragment was subcloned into KpnI/NcoI-digested d1EGFP plasmid (Clontech, d1EGFP = EGFP-Odc1) to yield 8xGliBS-EGFP-Odc1. The amino acid sequence of the Odc1-derived destabilizing element is: SHGFPPAVAAQDDGT LPMSCAQESGMDRHPAACASARINV.

#### 8xGliBS-EGFP-NLS-Odc1

cDNA encoding the polypeptide sequence SDPKKKRKVDPKK KRKVDPKKKRKVGYKKL, a triple tandem repeat of the SV40 large T antigen NLS, was assembled from the following four primers: 5′-GTACAAGTCCGATCCAAAAAAGAAGAGAAAG GTAGATCC-3′, 5′-AAAAAAGAAGAGAAAGGTAGATCCAAAAAAGAAGAGAAAGGTAGG-3′, 5′-GTACCCTACCTTTCTCTTCTTTTTTGGATCTACCTTT-3′, and 5′-CTCTTCTTTTTTGGATCTAC CTTTCTCTTCTTTTTTGGATCGGACTT-3′. The primers were treated with T4 polynucleotide kinase to phosphorylate the 5′ ends, mixed, melted at 95°C for 1 minute, annealed at 65°C for 1 minute, cooled briefly to room temperature, and then ligated into an 8xGliBS-EGFP-Odc1 vector previously digested with BsrGI and dephosphorylated with Antarctic phosphatase (NEB) to yield 8xGliBS-EGFP-NLS-Odc1.

#### 8xGliBS-IVS2-EGFP-NLS-Odc1

The IVS2 intron was PCR-amplified from pEF1a-IVS2-EGFP-polyA-Tol2 [38] using the primers: 5′-TTATGCTAGCGACCGATCCTGAGAACTTCAGG-3′ and 5′-TTATAGATCTCTTTGCCAAAATGATGAGACAGC-3′. The amplicon was then digested with NheI/BglII and ligated into NheI/BglII-cut 8xGliBS-EGFP-NLS-Odc1 to yield 8xGliBS-IVS2-EGFP-NLS-Odc1

#### 8xGliBS-IVS2-mCherry-NLS-Odc1

mCherry was PCR amplified from pRSET-mCherry [40] (kindly provided by Dr. Roger Tsien) using the primers: 5′-TTATAGATCTCCACCATGGTG AGCAAGGGCGAG-3′ and 5′-TTATTGTACACCTTGTACAGCTCGTCCATGCC-3′. The PCR product was digested with BglII/BsrGI and then ligated into BglII/BsrGI-digested 8xGliBS-IVS2-EGFP-NLS-Odc1 to yield 8xGliBS-IVS2-mCherry-NLS-Odc1.

#### MCS-polyA-Tol2

A multiple cloning sequence (MCS) was assembled with the primers: 5′-TCGAGGCTAGCGTCGACGAATTCCTGCAGAAGCTTGATATCGATGGATCCT and 5′-GTACA GGATCCATCGATATCAAGCTTCTGCAGGAATTCGTCGACGCTAGCC-3′ which was then ligated into XhoI/BsrGI-digested pT2KXIG [38] (kindly provided by Dr. Koichi Kawakami) to yield a vector containing the MCS flanked by Tol2 transposase recognition elements.

#### 8xGliBS-IVS2-mCherry-NLS-Odc1-polyA-Tol2

The 8xGliBS-IVS2-mCherry-NLS-Odc1 cassette was PCR-amplified from 8xGliBS-IVS2-mCherry-NLS-Odc1 using the primers: 5′-TAC TCGAGCGAGCTAACTTGTTTATTGCAGCT-3′ and 5′-ATGAATTCCTATCACTTCTTGTACCCT ACCTTTCTCTTCTT-3′. The amplicon was then digested with XhoI/EcoRI and ligated into XhoI/EcoRI-cut MCS-polyA-Tol2 to yield 8xGliBS-IVS2-mCherry-NLS-Odc1-polyA-Tol2.

#### 8xGliBS-IVS2-mCherry-NLS-polyA-Tol2

The 8xGliBS-IVS2-mCherry-NLS cassette was PCR-amplified from 8xGliBS-IVS2-mCherry-NLS using the primers: 5′-TACTCGAGCGAGCTAA CTTGTTTATTGCAGCT-3′ and 5′-ATGAATTCCTATCATACCTTTCTCTTCTTTTTTGGATCTAC -3′. The PCR product was then digested with XhoI/EcoRI and ligated into XhoI/EcoRI-cut MCS-polyA-Tol2 to yield 8xGliBS-IVS2-mCherry-NLS-polyA-Tol2.

The 8xGliBS-IVS2-mCherry-NLS-polyA-Tol2 and 8xGliBS-IVS2-mCherry-NLS-Odc1-polyA-Tol2 plasmids were maxiprepped, treated with proteinase K (10 µg DNA, 1 mg/mL proteinase K, and 0.5% SDS in a 50-µL reaction at 55°C for 30 minutes), subjected to a phenol/chloroform extraction (aqueous volume increased to 150 µL with water, extracted twice with 150 µL 25∶24∶1 neutral-buffered phenol∶chloroform∶isoamyl alcohol (Invitrogen), and then twice again with pure chloroform), and then precipitated with an equal volume of isopropanol after adjusting the solution pH to 4.5 with 0.3 M sodium acetate. Zebrafish zygotes were then co-injected with this RNAse-free plasmid DNA and *Tol2* transposase mRNA transcribed from pCS-TP [38] (kindly provided by Dr. Koichi Kawakami) using the Ambion SP6 mMessage mMachine kit (25 pg of each/embryo; 2-nL injection volume). The resulting adults were mated with wildtype AB fish to identify founders with germline transmission of the Hh pathway reporter, and those yielding F1 and F2 generations with robust, monoallelic reporter expression were used to establish transgenic colonies. Heterozygote *Tg(8xGliBS:mCherry-NLS)* and *Tg(8xGliBS:mCherry-NLS-Odc1)* lines in wildtype and mutant backgrounds were used in subsequent studies.

### Tail amputations

Adult caudal fins were amputated by first anesthetizing the zebrafish with 0.2 mg/mL tricaine in fish-system water for approximately 5 minutes. The fish were next placed onto a clean paper towel, and their tails were snipped using spring scissors (Fine Science Tools, Cat. No. 91501-09). The fish were then allowed to recover in system water.

### Transient genetic and molecular perturbations

Zebrafish *shha* mRNA was transcribed from a pSP64TS-shha plasmid [41] using the SP6 mMessage mMachine kit and then injected into zebrafish zygotes (150 pg/embryo). Cyclopamine (Infinity Pharmaceuticals) was dissolved in ethanol and applied to embryos at a final concentration of 100 µM, beginning at the 1-cell or 10-somite stages.

### Zebrafish imaging

Brightfield and fluorescence images were acquired using either a Leica DM4500B compound microscope equipped with a Retiga SRV camera or a Leica MZFLIII stereomicroscope with a Leica DFC480 camera. GFP and mCherry fluorescence was visualized using GFP and Texas Red filtersets, respectively. For live-imaging studies, zebrafish embryos were manually dechorionated, anesthetized in E3 medium containing 0.05% (w/v) tricaine mesylate, and then placed in agarose wells. Adult fish were similarly anesthetized but imaged on a dark surface.

## Results and Discussion

### Generation of zebrafish with nuclear Hh pathway reporters

**Figure 1:**
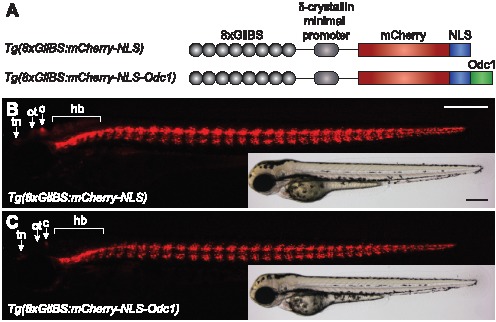
Generation of zebrafish with nuclear Hh pathway reporters. (A) Design of the Gli-dependent nuclear mCherry reporter constructs. (B–C) *Tg(8xGliBS:mCherry-NLS)* and *Tg(8xGliBS:mCherry-NLS-Odc1)* larvae at 84 hpf. Fluorescence and brightfield images of representative zebrafish are shown, and the telencephalic nuclei (tn), optic tectum (ot), cerebellum (c), and hindbrain (hb) are labeled. Embryo orientations: lateral view and anterior left. Scale bars: 200 µm.

To visualize *in vivo* Hh signaling with high detection sensitivity and single-cell resolution, we sought to generate transgenic zebrafish carrying Gli-dependent, nuclear-localized fluorescent reporters. Hh pathway-driven expression of exogenous genes in cultured cells or live organisms has been previously achieved using a minimal δ-crystallin promoter and eight tandem Gli binding sites derived from the murine Fox2A floor plate enhancer [39]. Therefore we prepared reporter constructs that coupled these regulatory elements with sequences encoding either mCherry-NLS alone or the nuclear-localized fluorescent protein tagged with an ornithine decarboxylase-derived destabilizing peptide [42] (mCherry-NLS-Odc1) (Figure 1A). We reasoned that the fast maturation kinetics [40] and discrete subcellular localization of mCherry-NLS reporter would allow the rapid detection of Hh pathway activation in individual cells; addition of the destabilizing domain would enhance reporter turnover and help reveal temporal changes in Gli function. Both constructs were individually cloned into a vector with flanking Tol2 transposase recognition elements, and the resulting plasmids were injected into zebrafish zygotes with *Tol2* mRNA.

Approximately 10–20% of adult zebrafish subjected to this transgenic procedure exhibited germline integration of the Gli-dependent reporter, and these F0 founders (both male and female) typically gave rise to F1 carriers with a transmission efficiency of 5%. For each transgenic line, three founders were chosen for further analysis, and we identified those yielding progeny with robust, Hh pathway-dependent reporter expression that segregated as a single insertion allele in the F2 generation. Based on these criteria, we maintained one line each for the *Tg(8xGliBS:mCherry-NLS)* and *Tg(8xGliBS:mCherry-NLS-Odc1)* transgenes.

**Figure 2:**
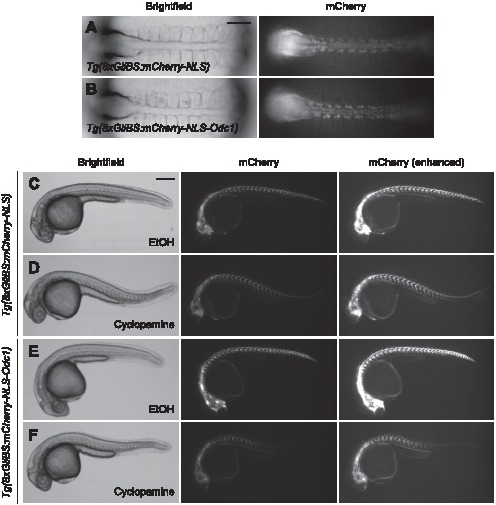
*Tg(8xGliBS:mCherry-NLS-Odc1)* zebrafish exhibit enhanced reporter turnover. (A–B) Brightfield and fluorescence images of 10-somite (∼14 hpf) *Tg(8xGliBS:mCherry-NLS)* and *Tg(8xGliBS:mCherry-NLS-Odc1)* embryos. (C–F) The transgenic lines at 30 hpf after the addition of cyclopamine or vehicle control at the 10-somite stage. The embryos were concurrently treated with phenylthiourea (0.003%, w/v) to block pigmentation. Brightness-enhanced fluorescence images are also shown to more clearly depict mCherry-positive cells in cyclopamine-treated embryos. The zebrafish embryos in (A) and (B) are the same animals shown in (D) and (F), respectively. Embryo orientations: A–B, dorsal view and anterior left; C–F, lateral view and anterior left. Scale bars: A–B, 100 µm; C–F, 200 µm.

Both reporter lines exhibited prominent mCherry fluorescence in the somites, ventral brain and neural tube, and other Hh-responsive tissues (Figure 1B–C). We observed some differences in reporter activity within the brain of larval-stage animals (84 hpf), likely due to the presence or absence of the Odc1-derived destabilizing element. Although cerebellar and telencephalic tissues exhibited reporter expression in the two transgenic lines, mCherry fluorescence was undetectable in the hindbrain and optic tectum of *Tg(8xGliBS:mCherry-NLS-Odc1)* larvae. These observations suggest that Hh morphogen-dependent patterning of these neural domains is largely complete by 84 hpf, where its actions within the cerebellum and telecephalon are ongoing. Differences between the two reporter lines could also be discerned at earlier stages upon addition of the Smo antagonist cyclopamine [43]. While mCherry fluorescence persisted in 30-hpf *Tg(8xGliBS:mCherry-NLS)* zebrafish previously exposed to cyclopamine for several hours, similarly treated *Tg(8xGliBS:mCherry-NLS-Odc1)* embryos lost mCherry signals in the ventroanterior brain, ventral spinal cord and posterior somites (Figure 2). Thus, the Odc1-derived destabilization domain increases Hh reporter turnover.

### Nuclear Hh pathway reporters convey cellular activity with improved sensitivity

**Figure 3:**
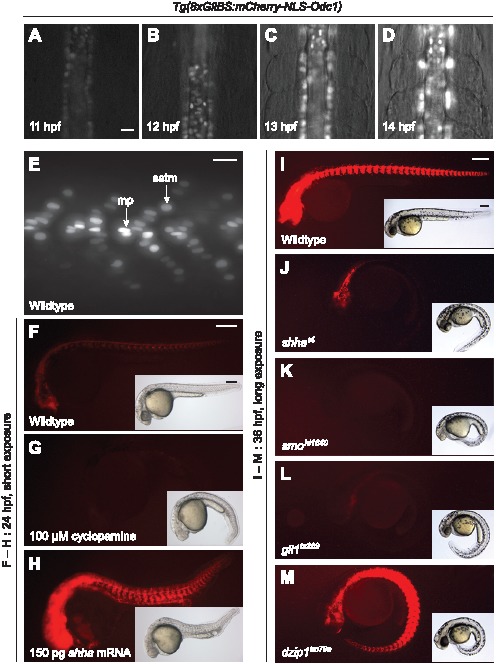
*Tg(8xGliBS:mCherry-NLS-Odc1)* zebrafish report Hh pathway activity with high sensitivity and fidelity. (A–D) Videomicroscopy frames of a *Tg(8xGliBS:mCherry-NLS-Odc1)* embryo during early somitogenesis. (E) Somitic region of a *Tg(8xGliBS:mCherry-NLS-Odc1)* zebrafish at 24 hpf, revealing cellular differences in nuclear mCherry fluorescence. Muscle pioneer cells (mp) and superficial slow-twitch muscle fibers (sstm) are labeled. (F–H) *Tg(8xGliBS:mCherry-NLS-Odc1)* zebrafish treated with cyclopamine or *shha* mRNA at the one-cell stage. Fluorescence and brightfield images of representative 24-hpf embryos are shown. (I–M) *Tg(8xGliBS:mCherry-NLS-Odc1)* zebrafish in wildtype or the indicated mutant backgrounds. Fluorescence and brightfield images of representative 36-hpf embryos are shown. The fluorescence micrographs were acquired with longer exposure times than those in panels F–H to enable the detection of low-level reporter activity. Brightfield micrographs show the distinctive *you*-type phenotypes observed within the same clutch of embryos. Embryo orientations: A–D, dorsal view and anterior up; E–M, lateral view and anterior left. Scale bars: A–E, 20 µm; F–M, 200 µm.

Due to the enhanced temporal resolution afforded by the *mCherry-NLS-Odc1* reporter, we focused on this transgenic line for further analysis of embryonic Hh pathway activity. Nuclear mCherry fluorescence could be first detected by the onset of somitogenesis (11 hpf), several hours earlier than in previous Hh reporter lines [15], [16]. Mesodermal adaxial cells and overlying neural plate cells in the midline exhibited reporter expression, which progressively increased during somite formation and differentiation (Figure 3A–D and Movie S1). Upon the completion of somitogenesis at 24 hpf, varying levels of mCherry fluorescence could be observed within myotome. Mononucleate fibers near the horizontal myoseptum exhibited the highest nuclear signals, and more distally positioned mononucleate fibers had mid-level reporter expression (Figure 3E). Multinucleated cells within the somitic mass did not express detectable levels of nuclear mCherry. These differences coincide with the locations of muscle pioneer cells and superficial slow-twitch muscle fibers, which respectively achieve high and intermediate thresholds of Hh pathway activity in response to notochord-derived morphogens [31]. Indeed, the lateral migration of mCherry-positive slow-twitch muscle precursors within each somite can be visualized through time-lapse videomicroscopy of *Tg(8xGliBS:mCherry-NLS-Odc1)* embryos (Movie S2). In contrast, the multinucleate fast-twitch muscle fibers that comprise the myotome bulk are not known to be Hh ligand-responsive [31]. Thus, physiologically important variations in Hh signaling can be discerned with single-cell resolution in the *Tg(8xGliBS:mCherry-NLS-Odc1)* line.

### Nuclear Hh pathway reporters respond to chemical and genetic perturbations

We next sought to confirm the fidelity of the *Tg(8xGliBS:mCherry-NLS-Odc1)* reporter by assessing its responsiveness to chemically and genetically induced changes in Hh pathway state. We first treated *Tg(8xGliBS:mCherry-NLS-Odc1)* zygotes with cyclopamine. Consistent with the resulting Hh loss-of-function phenotypes, including ventral body curvature and U-shaped somites [25], [26], mCherry fluorescence was undetectable in these fish (Figure 3F–G). We similarly observed that overexpression of Shha through mRNA injection caused strong mCherry upregulation throughout the embryo (Figure 3H).

As a third method to validate reporter fidelity, we analyzed *Tg(8xGliBS:mCherry-NLS-Odc1)* reporter output in mutant backgrounds with varying degrees of Hh pathway dysregulation, including the loss-of-function mutants *shha^t4^* [35], *smo^hi1640^* [26], and *gli1^ts269^* [36] (Figure 3I–L). We also assessed reporter activity in *dzip1^tm79a^* mutants [36] (Figure 3M), which exhibit decreased Hh target gene expression in the ventral brain and neural tube but increased pathway activation in somitic tissues [44], [45]. Consistent with the morphological and molecular phenotypes associated with these mutant alleles, we observed tissue-specific changes in reporter expression. Embryos lacking Smo function resembled cyclopamine-treated fish and were essentially devoid of detectable mCherry fluorescence. In comparison, *shha^t4^* and *gli1^ts269^* mutants lacked mCherry signals within the myotome but maintained a reduced reporter activity in anterior tissues, and *dzip1^tm79a^* mutants upregulated and downregulated Hh reporter expression within the somites and ventral brain, respectively. Taken together, our observations demonstrate that the Gli-dependent nuclear reporters are sensitive, specific indicators of Hh pathway state.

### Nuclear Hh pathway reporters function in adult zebrafish

We concluded our studies by investigating the functionality of our Hh pathway reporters in adult zebrafish. Caudal fin regeneration is a convenient model of Hh pathway-dependent physiology; previous studies have established clear domains of Hh ligand production and response during this process [32], and fin structures are particularly amenable to live imaging. After fin amputation, epithelial cells rapidly cover the wound and a blastema of de-differentiated cells forms within each bony ray segment [46], [47]. The blastema then proliferates and gives rise to daughter cells that reconstitute the fin in an epimorphic manner. During this regenerative process, *shha* is initially expressed around 30 hours post amputation (hpa) in a subset of basal epidermal cells at distal end of each ray [32]. Shha-responsive *ptch2*-positive cells can subsequently be detected at 40 hpa in the epidermis, first overlapping with the distal *shha*-positive domain and then extending proximally [32]. These signaling events regulate dermal bone patterning as the ray segments reform from blastema-derived cells.

**Figure 4:**
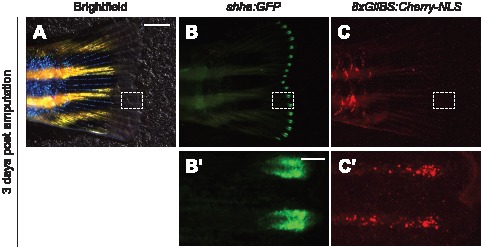
Visualization of Hh signaling during adult fin regeneration. Caudal fin of a *Tg(-2.4shha:GFP-ABC;8xGliBS:mCherry-NLS)* zebrafish three days after amputation. Brightfield (A) and fluorescence micrographs (B–C) are shown, with the dashed boxes corresponding to the magnified views below. Fin orientations: lateral views, anterior left. Scale bars: A–C, 2 mm; B′–C′, 200 µm.

To simultaneously visualize *shha*-producing cells and their *ptch2*-expressing responders, we crossed *Tg(-2.4shha:GFP-ABC)* [37] and *Tg(8xGliBS:mCherry-NLS)* zebrafish to obtain progeny carrying both fluorescent reporters. Shha-expressing cells in these transgenic organisms are labeled with GFP, and the corresponding cellular responses can be assessed by mCherry fluorescence. We cut the caudal fins of adult *Tg(-2.4shha:GFP-ABC;8xGliBS:mCherry-NLS)* zebrafish and monitored changes in GFP and mCherry reporter expression at 3 days post amputation. As expected, amputation caused a dramatic increase in GFP expression at the distal end of each fin ray (Figure 4A–B), and mCherry-expressing cells populated interray regions immediately proximal to the GFP-positive domains (Figure 4C).

### Conclusion

Taken together, our results demonstrate the utility of nuclear-localized mCherry reporters of Hh pathway activity, particularly when used in conjunction with optically transparent model organisms such as the zebrafish. The *Tg(8xGliBS:mCherry-NLS)* and *Tg(8xGliBS:mCherry-NLS-Odc1)* fish described here have certain advantages over previous Hh pathway reporter lines, including: (1) higher detection sensitivity that enables real-time observation of Hh signaling by the onset of somitogenesis; and (2) greater cellular resolution that allows pathway activity differences between neighboring cells to be discerned. We anticipate that these transgenic organisms will be valuable tools for studying the Hh pathway-dependent processes that contribute to embryonic development, tissue homeostasis, and tumorigenesis. They could also facilitate the identification of new genetic and chemical modulators of Hh signal transduction through high-content, image-based screens.

## Supporting Information

Movie S1 **Real-time imaging of Hh signaling during early somitogenesis.** Nuclear mCherry expression in a *Tg(8xGliBS:mCherry-NLS-Odc1)* zebrafish between 11 and 15 hpf. The midline region between the future sixth and ninth somites was imaged at a rate of 1 frame/minute, and the movie is shown at rate of 30 frames/second. Embryo orientation: dorsal view and anterior up. Field of view: 180 µm×180 µm.
(MOV)

Movie S2 **Real-time imaging of slow-twitch muscle precursor migration.** Nuclear mCherry expression in a *Tg(8xGliBS:mCherry-NLS-Odc1)* zebrafish between 13 and 17 hpf. The midline region between the second and seventh somites was imaged at a rate of 1 frame/minute, and the movie is shown at rate of 30 frames/second. Embryo orientation: dorsal view and anterior up. Field of view: 260 µm×260 µm.
(MOV)
